# Field-Caught Permethrin-Resistant *Anopheles gambiae* Overexpress CYP6P3, a P450 That Metabolises Pyrethroids

**DOI:** 10.1371/journal.pgen.1000286

**Published:** 2008-11-28

**Authors:** Pie Müller, Emma Warr, Bradley J. Stevenson, Patricia M. Pignatelli, John C. Morgan, Andrew Steven, Alexander E. Yawson, Sara N. Mitchell, Hilary Ranson, Janet Hemingway, Mark J. I. Paine, Martin J. Donnelly

**Affiliations:** 1 Vector Group, Liverpool School of Tropical Medicine, Liverpool, United Kingdom; 2 Biotechnology and Nuclear Agricultural Research Institute, Ghana Atomic Energy Commission, Kwabenya, Accra, Ghana; Princeton University, Howard Hughes Medical Institute, United States of America

## Abstract

Insects exposed to pesticides undergo strong natural selection and have developed various adaptive mechanisms to survive. Resistance to pyrethroid insecticides in the malaria vector *Anopheles gambiae* is receiving increasing attention because it threatens the sustainability of malaria vector control programs in sub-Saharan Africa. An understanding of the molecular mechanisms conferring pyrethroid resistance gives insight into the processes of evolution of adaptive traits and facilitates the development of simple monitoring tools and novel strategies to restore the efficacy of insecticides. For this purpose, it is essential to understand which mechanisms are important in wild mosquitoes. Here, our aim was to identify enzymes that may be important in metabolic resistance to pyrethroids by measuring gene expression for over 250 genes potentially involved in metabolic resistance in phenotyped individuals from a highly resistant, wild *A. gambiae* population from Ghana. A cytochrome P450, *CYP6P3*, was significantly overexpressed in the survivors, and we show that the translated enzyme metabolises both alpha-cyano and non–alpha-cyano pyrethroids. This is the first study to demonstrate the capacity of a P450 identified in wild *A. gambiae* to metabolise insecticides. The findings add to the understanding of the genetic basis of insecticide resistance in wild mosquito populations.

## Author Summary

Malaria, a disease spread by anopheline mosquitoes, is a global health problem with an enormous economic and social impact. Pyrethroid insecticides are critical in reducing malaria transmission, and resistance to these insecticides threatens current control efforts. With a limited number of public health insecticides available for the foreseeable future, it is vital to monitor levels of resistance to facilitate decisions on when new strategies should be implemented before control fails. For monitoring, simple molecular assays are highly desirable, because they can detect resistance at very low frequencies and should identify the presence of single recessive alleles well before bioassays. An understanding of the mechanisms conferring resistance facilitates the development of such tools and may also lead to novel strategies to restore the efficacy of the insecticide, or the development of new compounds. We set out to identify enzymes that may confer metabolic pyrethroid resistance by comparing levels of messenger RNA between insecticide-selected versus unselected mosquitoes. We caught members of the major malaria vector, *A. gambiae s.s.* from a highly pyrethroid resistant field population. We found increased transcript levels for a cytochrome P450, *CYP6P3*, and demonstrate that it encodes for an enzyme that metabolises pyrethroids.

## Introduction

Insecticide resistance in disease vectors is one of the greatest challenges to the reduction of the burden caused by vector-borne diseases in developing countries. Beyond their public health importance insect vectors are increasingly regarded as model organisms with insecticide resistance serving as an excellent example of natural selection [1]. In many parts of Africa, the malaria vector, *Anopheles gambiae* shows high levels of resistance to pyrethroid insecticides which are the mainstay of vector control [2] and there is evidence that this resistance may reduce the efficacy of treated bednets and indoor residual spraying with pyrethroids [3]. Moreover, some pyrethroid resistance mechanisms that confer cross-resistance to DDT are geographically widespread [4]. The stark reality facing control program managers is that there is resistance to the primary compounds used for vector control and that no new active ingredients have become commercially available for public health use in the last 20 years. Studies of resistance mechanisms are key to both understanding the evolution of resistance and to minimising its impact on disease control.

At the biochemical level, two classes of mechanism are predominantly associated with insecticide resistance; changes in the sensitivity of insecticide targets in the nervous system and metabolism of insecticides before they reach their target [5]. In *A. gambiae*, target-site resistance to DDT and pyrethroids is associated with a knock-down resistance (*kdr*) mutation in the voltage-gated sodium channel gene. The *kdr* alleles are characterised by two point mutations resulting in either a L1014F [6] or L1014S [7] substitution. The mutations may be present alone or in combination and have arisen from multiple mutation events [1].

While insecticide resistance associated with *kdr* is well studied at the physiological, behavioural and population level, much less is known about the enzymes associated with metabolic resistance. One route of metabolic resistance is through up-regulation of detoxification enzymes. Overexpression of enzymes related to insecticide resistance is generally assumed to be associated with cytochrome P450-dependent monooxygenases (P450), carboxylesterases (COE), and glutathione-S transferases (GST). Among these three families, evidence suggests that P450s commonly play a primary role in pyrethroid resistance (for reviews see [8] and [9]). To date, out of 111 putative *A. gambiae s.s.* P450s [10] four have been observed to be overexpressed in adult mosquitoes from colonies characterised as pyrethroid resistant, namely *CYP6Z1*, *CYP6Z2*, *CYP6M2* and *CYP325A3* [11]–[13]. Although the up-regulation of these P450s was associated with resistance, their potential to metabolise pyrethroids remains unclear. While Chiu *et al.* [14] demonstrated that CYP6Z1 metabolises DDT, to date only CYP6Z2 interacts with pyrethroids. McLaughlin *et al.* [15] found that CYP6Z2 binds to two pyrethroids, permethrin and cypermethrin. Their data, however, also suggested that the pyrethroids were not metabolised by this P450 highlighting the importance of functionally characterising putative candidates involved in pyrethroid metabolism. These earlier studies have potential confounding effects of colonisation including genetic drift and physiological adaptations to the artificial laboratory environment. The study of immune response in natural mosquito populations has highlighted that mechanisms found in laboratory colonised material may be less relevant in nature [16].

While genetic markers for target site insensitivity are available [6],[7] and widely used, we lack simple screening methods for alleles associated with up-regulation of detoxification enzymes. As a result, the role of metabolic resistance in reducing the efficacy of malaria vector control is unknown. The current study was carried out as part of the Innovative Vector Control Consortium (IVCC) to develop a tool to monitor mosquito field populations for resistance alleles [17]. We set out to identify enzymes that metabolise pyrethroid insecticides by selecting field-caught mosquitoes against the lethal time to kill 50% of the mosquito population (LT_50_). Genes potentially associated with detoxification of xenobiotics were screened for differential gene expression between survivors and unexposed mosquitoes using the *A. gambiae detox chip* [11]. We then expressed the most promising candidate in *Escherichia coli* to examine its pyrethroid metabolising potential.

## Materials and Methods

### Mosquito Collections

Mosquito collections were carried out in the village of Dodowa, Ghana (05°52.67′N, 000°06.36′W) between October and November 2006. A detailed description of the field site can be found in Yawson *et al.* [18]. Mosquitoes morphologically identified as members of the *A. gambiae* species complex [19],[20] were sampled from natural breeding sites and raised to adults in an insectary located in Dodowa. Larvae were given ground TetraMin fish food and adults were provided with 10% sugar solution. Newly emerged adults were separated into females and males and kept as cohorts of same age. All bioassays and selections were performed on the third day post-eclosion.

In addition to the larval collections blood-fed females were caught using aspirators inside houses and family lines reared as described in Müller *et al.* [21]. These family lines were used to compare constitutive versus induced gene expression (see below).

### Selection Experiment

Before selecting mosquitoes against permethrin we determined the lethal time (LT) of 0.75% permethrin treated filter paper for 50% mortality (LT_50_) using World Health Organization (WHO) test kits following standardised conditions [22]. To estimate the LT_50_ we first established a time-response curve by exposing approximately 100 individuals to one of six different exposure times (12.5, 25, 50, 100, 150 and 200 min). Mortality was recorded 24 h post exposure and data were fitted by a logistic regression model using logit-transformed probabilities [23] to predict the LT_50_. All analyses were performed using the open source statistical software package R (http://www.r-project.org). All R-code required to perform these calculations is available from the first author on request. Once the LT_50_ was determined, cohorts of 3-day old adult females were split into two groups; one group was exposed to 0.75% permethrin and the other group to a control paper which contained only the insecticide carrier (silicone oil). Both groups were exposed for the LT_50_ using WHO test tubes and then transferred to holding tubes. In order to examine constitutive differences in gene expression between selected and unselected mosquitoes all individuals were kept in the holding tubes for 48 h before they were killed in 70% ethanol. A recovery time of 48 h was chosen to control for potential permethrin-induced gene expression. Vontas *et al.* [24] showed that permethrin-induced gene expression regains constitutive levels within 24 h of a non-lethal exposure. To test for permethrin-induced gene expression additional family lines were reared and 3-day old adult females split into two groups. One group was exposed to 0.75% permethrin for 30 min and the second group served as a control. After a recovery time of 48 h post exposure four to five female mosquitoes from each group were pooled and RNA extracted. Using RT-PCR gene expression levels of exposed and unexposed individuals were compared in a pair wise *t*-test.

For all mosquitoes one hind leg was removed for DNA extraction and the remaining body parts were transferred to RNA*later* (Ambion) to prevent RNA degradation. Genomic DNA was extracted from legs using the DNeasy kit (Qiagen) and used to identify each specimen to species and molecular form [25]. The same DNA was used to screen for the presence of the L1014F [6] and L1014S [7] substitutions within the voltage-gated sodium channel protein causing knockdown resistance (*kdr*) by a heated oligonucleotide ligation assay (HOLA) [26].

### Microarray

Only mosquitoes identified as members of *A. gambiae s.s.* S form and homozygous for the L1014F *kdr* allele were included in the microarray study. Total RNA was extracted from pools of 10 mosquitoes which were either selected against 0.75% permethrin for the LT_50_ or not exposed to the insecticide. The quality and quantity of all RNA pools was measured by a spectrophotometer (Nanodrop Technologies) and a random subset was also assessed using a 2100 Bioanalyzer (Agilent Technologies). RNA extraction, amplification and labelling protocols followed those described in Müller *et al.* [21]. Labelled targets were hybridised to an updated version of the *A. gambiae detox chip* [11],[21] which was printed with a physical rearrangement of the probes (ArrayExpress accession A-MEXP-863). The probes on the microarray include 103 cytochrome P450s, 31 esterases, 35 glutathione S-transferases and 85 additional genes such as peroxidases, reductases, superoxide dismutases, ATP-binding cassette transporters, tissue specific genes and housekeeping genes.

The microarray experiment compared RNA pools from selected vs. unselected mosquitoes, comprising six independent replicates with dye-swaps (12 arrays in total). As each probe was spotted in replicates of four and measurements were obtained for both red and green wavelengths in each array, a total of 96 measurements per probe were obtained. After visual inspection of each array, spot and background intensities were calculated from the scanned array images using GenePix Pro 5.1 software (Axon Instruments). Raw intensities were then analysed with Limma 2.4 software package [27] running in R. Any spot that showed a median intensity in one or both channels at saturation was excluded from the analysis. For each spot background intensities were subtracted (i.e. method = “subtract”) from the total spot intensities and adjusted intensities were transformed into intensity log-ratios and normalised. For the comparison between the two groups, selected vs. unselected, estimates for technical replicates (dye-swaps) were first averaged and then compared between the two groups. A detailed description of the methods used for normalisation and statistical analysis is given in Müller *et al.* [12]. All microarray data has been deposited in ArrayExpress (accession E-MTAB-52).

In terms of absolute fold change our values are likely to underestimate true fold differences between mosquitoes that would survive an LT_50_ and those that would not. This is a result of the study design whereby the LT_50_ survivors were compared with a control group that would be expected to be a mixture of 50% mosquitoes surviving and 50% mosquitoes dying after exposure to 0.75% permethrin. It was not possible to select a fully susceptible control group due to the expected RNA degradation postmortem. The underestimation of fold changes may occur wherever resistant mosquitoes are compared with their parental line. Details of how this study design limits maximum fold change are given in Figure S1. As a consequence we have chosen to rank our genes by statistical significance (*i.e.*, −log_10_ *P*-value) rather than setting an arbitrary fold change cut-off to filter for candidates.

### Quantitative RT-PCR

Quantitative RT-PCR was used to validate microarray data and for comparisons with the “Kisumu” strain, a susceptible *A. gambiae s.s.* laboratory colony. An aliquot of 75 ng from each pool of total RNA served as template for making target specific cDNA by reverse transcription in a single multiplex assay using the GenomeLab GeXP Start Kit (Beckman Coulter) and the gene-specific primers in Table 1. The primers were designed using the eXpres Profiler software (Beckman Coulter) based on cDNA sequences retrieved from the sources given in Table 1. The GeXP multiplex system uses a combined primer of target-specific and a universal sequence to reverse transcribe mRNA into cDNA. The reverse transcription step was followed by a PCR step in which during the first three cycles amplification was carried out by chimerical forward and reverse primers (Table 1). For the subsequent cycles (numbers 4 to 35), amplification was carried out using universal forward and universal reverse primers provided by the kit. The PCR conditions were 95°C for 10 min, followed by 35 cycles of 94°C for 30 s, 55°C for 30 s and 68°C for 1 min. Multiplexing primer specificity was confirmed by sequencing the PCR products obtained from single reactions. The universal primers that come with the kit were fluorescently labelled and yielded signals that corresponded to the amount of product in the multiplex reaction. PCR products were quantified with a CEQ 8000 Genetic Analysis System (Beckman Coulter) running a GenomeLab GeXP eXpress analysis program (Beckman Coulter) that computes peak areas for each target. The peak area of a control gene, *S7* (VectorBase: AGAP010592) was used to normalise for variation in total mRNA amount. Normalised peak areas were then log_2_-transformed to approximate a normal distribution.

**Table 1:** Oligonucleotide primer sequences used for microarray validation.

Gene	Accession no.	Primer	Sequence (5′-3′)	Concentration	Size
*ABCC11*	VectorBase:AGAP008436-RA	forward	TCATCTACCGGGACTTTTCG	20 nM	135 bp
		reverse	TCCCAATGAAGCTGGATTTC	50 nM	
*ABCC9*	VectorBase:AGAP008437-RA	forward	AACGTCCACACCGATCTTTC	20 nM	106 bp
		reverse	TTCCAATCGCTTTAATTGCC	50 nM	
*COEAE2G*	VectorBase:AGAP006723-RA	forward	TGATCAAGAACCTGTCGGTG	20 nM	177 bp
		reverse	CGGTAAGCAGATCGACCAAT	150 nM	
*CYP12F4*	VectorBase:AGAP008018-RA	forward	GGATCGACGGGAATTCTGTA	20 nM	215 bp
		reverse	AGAACGAGGTCTTTTCCGGT	50 nM	
*CYP4D22*	VectorBase:AGAP002419-RA	forward	GTTAGCGTTGTTCTGCACCA	20 nM	184 bp
		reverse	GATCTTGAAGTGAAAGGCGG	50 nM	
*CYP4H19*	VectorBase:AGAP000088-RA	forward	TTCTCGTGACGCTATTGGTG	20 nM	238 bp
		reverse	CTGGTTACGACGACCATGTG	150 nM	
*CYP4H24*	VectorBase:AGAP000088-RA	forward	CGCAAGTGTCTAACGAGCAG	20 nM	163 bp
		reverse	TCATGACCCTCGAACATGAA	50 nM	
*CYP6AK1*	VectorBase:AGAP010961-RA	forward	GCTGCCACCTTCTATATGGC	20 nM	142 bp
		reverse	TTTCGCGTCCATATTTGACA	6.2 nM	
*CYP6M2*	VectorBase:AGAP008212-RA	forward	TTCGTCGACTCTCCTCACCT	20 nM	199 bp
		reverse	GAAATGTACCGGGACTGGTG	50 nM	
*CYP6M3*	VectorBase:AGAP008213-RA	forward	GATCAAGTACCGGGTGGAGA	20 nM	229 bp
		reverse	TCTGCCCTTATCTTGCACCT	24.4 pM	
*CYP6N1*	GeneBank:AY028786	forward	CTACTGGGAAAAGCGAGGTG	20 nM	149 bp
		reverse	GAATTCCTCCGAATGGTTGA	50 nM	
*CYP6P3*	VectorBase:AGAP002865-RA	forward	AGCTAATTAACGCGGTGCTG	20 nM	121 bp
		reverse	AAGTGTGGATTCGGAGCGTA	50 nM	
*CYP6Z2*	VectorBase:AGAP008218-RA	forward	TTATTTGTCCTGGGTTGTTGAA	20 nM	244 bp
		reverse	GTTTCTGCACCGGCAATGTA	50 nM	
*GSTD1-4*	VectorBase:AGAP004164-RC	forward	TCGAGCGATCATGTGCTATC	20 nM	222 bp
		reverse	AACGCTAAAGCTTCCCCAAT	50 nM	
*S7*	VectorBase:AGAP010592-RA	forward	CATTTCGTTGTGAACCCAAA	20 nM	128 bp
		reverse	AGTTCATCTCCAGCTCCAGG	0.8 nM	

Primer sequences and product size are given without the universals needed for the qPCR method applied.

### Cloning *CYP6P3* for Expression in *Escherichia coli*

Messenger RNA from the susceptible lab colony *A. gambiae* “Kisumu” (3-day old adults) was isolated using the PicoPure kit (Arcturus) and cDNA prepared using Superscript III (Invitrogen). Initial efforts to express *CYP6P3* using the *E. coli* OmpA signal peptide as previously described for *CYP6Z2* [15] were unsuccessful. Therefore, we used another common strategy for P450 expression, which is to replace the natural P450 amino-terminus with a sequence (MALLLAVF) derived from the bovine steroid 17 α-hydroxylase [28]. To introduce the amino-terminal 17α modification the 5′-end of *CYP6P3* cDNA was amplified using KOD DNA polymerase (Novagen) with ECG169 (5′TTTCATATGGCTCTGTTATTAGCAGTTTTTGCCGCGTTCATCTTCGCAGTGTCGATCGTG 3′), introducing a *Nde*I restriction site at the initiation codon (underlined), and ECG137 (5′-ATGAATTCTACAACTTTTCCACCTTCAAG -3′) complementary to the 3′-end of the *CYP6P3* cDNA, and introducing an *Eco*RI site (underlined). The resulting 17α-CYP6P3 was ligated into pCWompA2 via *Nde*I and *Eco*RI to create pCW::17α-cyp6p3. The construct was sequenced and compared with the database sequence of *CYP6P3* (GenBank:AAL93295). In addition to the four amino acid substitutions to the membrane anchoring sequence as a result of the 17α modification (E2A, I4L, N5L, and L8F – numbering relative to published sequenced), there were two nucleotide changes that encoded amino acid substitutions R154W and L292V. These nucleotides changes were are also present in *CYP6P3* amplified from Kisumu genomic DNA and are therefore not due to PCR errors.

### Preparation of *E. coli* Membranes for Functional CYP6P3

For functional expression of CYP6P3 and its redox partner cytochrome P450 reductase (CPR), competent *E. coli* DH5α cells were co-transformed with pCW::17α-cyp6p3 and pACYC-AgCPR. This transformant was grown in 0.4 l of Terrific Broth with ampicillin and chloramphenicol selection at 37°C until the optical density at 595 nm reached 0.8 units. The culture was then cooled to 25°C, supplemented with 0.5 mM 5-aminolevulinic acid (Melford, UK) and 1 mM isopropyl β-D-1-thiogalactopyranoside (Melford) before incubation continued at 25°C with orbital shaking at 150 rpm. The cells were harvested and membranes prepared as described previously [15]. P450 function was quantified by CO-difference spectroscopy [29] and CPR activity was estimated by cytochrome *c* reductase activity [30]. CYP6P3 was expressed at 50–100 nmol of P450 litre of culture. The isolated bacterial membranes contained 0.5 nmol of CYP6P3 per mg protein and the specific activity of *CPR* was 61 nmol cytochrome c reduced min^−1^ mg^−1^ protein. Total protein concentration was determined by Bradford assay, with bovine serum albumin standards.

### Pyrethroid Metabolism Assays

Deltamethrin and permethrin (Chemservice, West Chester, PA) were incubated with 0.25 µM CYP6P3 in 0.2 M Tris.HCl, pH 7.4, 0.25 mM MgCl_2_ in the presence or absence of an NADPH generating system (1 mM glucose-6-phosphate (Melford), 0.1 mM NADP^+^ (Melford), 1 unit ml^−1^ glucose-6-phosphate dehydrogenase (G6PDH) in a total volume of 100 µl. Reactions were carried out in triplicate at 30°C with 1,200 rpm shaking. Samples were pre-warmed for 5 min before reactions were initiated by addition of the membrane preparation. Reactions were stopped with 100 µl of acetonitrile and incubated for a further 20 min to ensure that all pyrethroid was dissolved.

The quenched reactions were centrifuged at 20,000 *g* for 10 min before transferring the supernatant to glass HPLC vials. 100 µl of the supernatant was loaded onto a mobile phase with a flow rate of 1 ml min^−1^ and 23°C for separation on a 250 mm C18 column (Acclaim 120, Dionex). Time-trial reactions were run with a linear gradient from 0% to 90% acetonitrile in water (v/v) over the first 6 min, 90% was then held for 10 min before returning 0% with a linear gradient over 2 min followed by equilibration with 0% for another 4 min. Pyrethroid elution was monitored by absorption at 232 nm and quantified by peak integration (Chromeleon, Dionex).

For kinetics of deltamethrin, varying concentrations of substrate (0.5–16 µM) were used. Deltamethrin concentrations were determined by HPLC as describe above, but using an isocratic mobile phase with 90% acetonitrile in water. Rates of deltamethrin turnover from three independent reactions were plotted versus deltamethrin substrate concentration. *K*_m_ and V_max_ were determined using SigmaPlot v10.0 (Systat Software, Inc) by fitting to the Michaelis-Menton equation using non-linear regression.

## Results

### Spectrum of Permethrin Susceptibility

**Figure 1:**
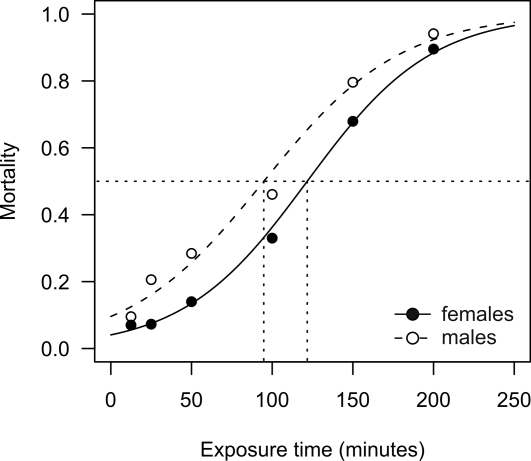
Spectrum of permethrin susceptibility in *A. gambiae s.l.* from Dodowa, southern Ghana. Proportion of 3-day old adult *A. gambiae s.l.* individuals killed as a function of exposure time to 0.75% permethrin following WHO standard protocols [22]. Dots represent summaries from performed susceptibility tests with between 98 and 110 individuals per time point and sex. Time-response curves were fitted to data using logit analysis [23]. Dotted lines indicate LT_50_s which were 2 h 2 min and 1 h 35 min for females and males, respectively.

Before selecting individuals the LT_50_ to 0.75% permethrin was determined by exposing 98 to 110 individuals per time point and sex (Figure 1). Using logistic regression models we estimated an LT_50_ of 122 min for females and 95 min for males. Mortality rates for a WHO standard 1 h exposure were 16.8% and 30.5% for females and males, respectively (Figure 1). Mortality in the controls was 2.3% for females (*N* = 171) and 2.1% for males (*N* = 116).

### Species Composition and Genotypes

A total of 333 *A. gambiae s.l.* females were stored for gene expression studies and identified to species level, molecular form and *kdr* genotype (Table 2). The majority (99.4%) of the sampled mosquitoes were *A. gambiae s.s.* belonging to the molecular S form, and only two individuals in the control group were M form. The L1014S *kdr* mutation was detected in three individuals, although it was not possible to confirm this result by sequencing. There was no difference in L1014F frequencies between the control and selected groups (Fisher's exact test, *P* = 0.22). This latter mutation was close to fixation with 91.3% of the screened individuals in the control group being homozygous for the L1014F mutation (Table 2).

**Table 2:** Distribution of S and M molecular forms and *kdr* allele frequencies in the control and selected group collected for gene expression analysis.

Molecular form	*kdr* allele frequency					
	R_W_,R_W_	R_W_,+	R_W_,R_E_ 1	R_E_,R_E_	R_E_,+	+,+
*Control group* (172)						
S	91.3% (157)	5.8% (10)	1.7% (3)	-	-	-
M	-	-	-	-	-	1.2% (2)
*Selected group* (161)						
S	95.0% (153)	5.0% (8)	-	-	-	-
M	-	-	-	-	-	-

Figures in brackets show the number of specimens scored.

R_W_: L1014F *kdr* substitution.

R_E_: L1014S *kdr* substitution.

1 not confirmed by sequencing.

### Gene Expression

**Figure 2:**
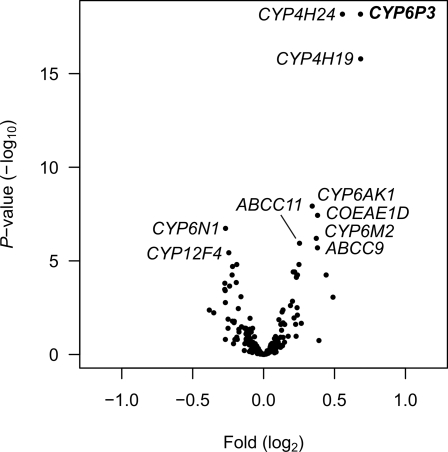
Microarray analysis of loci showing differences in expression levels between LT_50_-selected and unselected specimens. Each dot represents the mean estimates, *P*-value *vs.* fold difference for one unique probe on the microarray. Names are given for the 10 statistically most significant genes (values are given in Table 3). To account for multiple testing, *P*-values were adjusted adopting the approach of Benjamini and Hochberg [41] to control for the false discovery rate as described in Smyth [27].

All specimens included in the microarray analysis were *A. gambiae s.s.*, molecular S form and homozygous for the L1014F *kdr* mutation to minimise confounding effects. Three P450s were consistently (very low *P*-values) expressed at higher levels in LT_50_-selected vs. unexposed mosquitoes; *CYP6P3*, *CYP4H24* and *CYP4H19* (Figure 2, Table 3). *CYP6P3* and *CYP4H19* were 1.6-fold over-expressed and *CYP4H24* was 1.5-fold over-expressed in specimens surviving the LT_50_.

**Table 3:** Microarray results of top ten differentially expressed genes between selected and unselected mosquitoes.

Gene	Accession no.	Function	Location	Measured fold	Putative fold^1^	*P*-value^2^
Overexpressed in selected mosquitoes						
*CYP6P3*	VectorBase: AGAP002865-RA	Cytochrome P450	2R	1.61	2.82	6.69×10^−19^
*CYP4H24*	VectorBase: AGAP000088-RA	Cytochrome P450	X	1.47	2.21	6.69×10^−19^
*CYP4H19*	VectorBase: AGAP000088-RA	Cytochrome P450	X	1.61	2.83	1.63×10^−16^
*CYP6AK1*	VectorBase: AGAP010961-RA	Cytochrome P450	3L	1.27	1.57	1.19×10^−08^
*COEAE1D*	VectorBase: AGAP005756-RA	Carboxylesterase	2L	1.30	1.66	3.71×10^−08^
*CYP6M2*	VectorBase: AGAP008212-RA	Cytochrome P450	3R	1.29	1.64	6.28×10^−07^
*ABCC11*	VectorBase: AGAP008436-RA	ABC transporter	3R	1.19	1.38	1.15×10^−06^
*ABCC9*	VectorBase: AGAP008437-RA	ABC transporter	3R	1.30	1.66	2.01×10^−06^
Overexpressed in unselected mosquitoes						
*CYP6N1*	GenBank: AY028786	Cytochrome P450	3R	−1.20	−1.35	1.84×10^−07^
*CYP12F4*	VectorBase: AGAP008018-RA	Cytochrome P450	3R	−1.19	−1.32	3.67×10^−06^

1 Gives the estimated ratio in gene expression levels if survivors were directly compared with dead mosquitoes (See Figure S1 for its calculation).

2 To account for multiple testing, *P*-values were adjusted adopting the approach of Benjamini and Hochberg [41] to control for the false discovery rate as described in Smyth [27].

**Figure 3:**
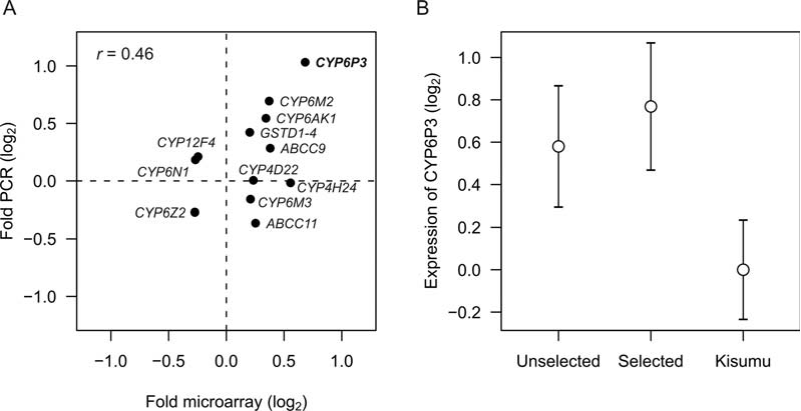
Microarray validation by quantitative RT-PCR. (A) Correlation between microarray data and RT-PCR of selected genes given in Table 1 (*CYP4H19* and *COEAE2G* were removed from the analysis). While both microarray and multiplex RT-PCR showed similar fold differences for *CYP6P3*, the overall correlation was weak (*r* = 0.46, *P* = 0.129). (B) *CYP6P3* expression levels including additional specimens (Mean values±S.E.M.). Unselected: unselected field specimens (*N* = 14 replicates, *n* = 140 individuals). Selected: LT_50_-selected specimens (*N* = 15 replicates, *n* = 150 individuals). Kisumu: susceptible lab strain (*N* = 3 replicates, *n* = 30 individuals). *CYP6P3* levels in the susceptible Kisumu lab strain were 1.7-fold lower than in the selected, field-caught mosquitoes (one-sided *t*-test, *P*<0.05). Note that the *y*-axis shows normalised, log-transformed expression levels.

The same RNA pools used in the microarray analysis were additionally evaluated by multiplex quantitative reverse transcription (RT) PCR for 14 selected genes (Table 1). The transcripts were selected from the pool of genes that were differentially expressed in the microarray analysis. Two genes, *CYP4H19* and *COEAE2G* were removed from the analysis due to missing PCR products for some of the RNA pools. Both methods were in concordance for several genes, though not for all, including *CYP6P3*, *CYP6M2*, *CYP6AK1*, *GSTD1-4*, *ABCC9* and *CYP6Z2* (Figure 3A). For all other genes, *ABCC11*, *CYP4D22*, *CYP4H24*, *CYP6M3*, *CYP6N1* and *CYP12F4* the lack of concurrence between the two methods is probably related to low levels of fold change [31].

On the basis of having the most consistent gene expression pattern, the catalytic properties of CYP6P3 enzyme was further investigated by heterologous expression in *E. coli*. The comparison of *CYP6P3* expression levels between LT_50_-selected mosquitoes and a susceptible laboratory (Kisumu, *A. gambiae s.s.*) strain also showed increased levels in the wild mosquito population (Figure 3B), showing additional evidence for an association between *CYP6P3*-overexpression and permethrin resistance. A comparison of *CYP6P3* expression levels between permethrin-exposed and unexposed female siblings 48 h post exposure showed no sign of gene induction (pair wise *t*-test, *P*-value = 0.49, *N* = 7 families; data not shown). Hence, overexpression of *CYP6P3* may be assumed to be constitutive rather than induced upon permethrin exposure.

### Pyrethroid Metabolism

**Figure 4:**
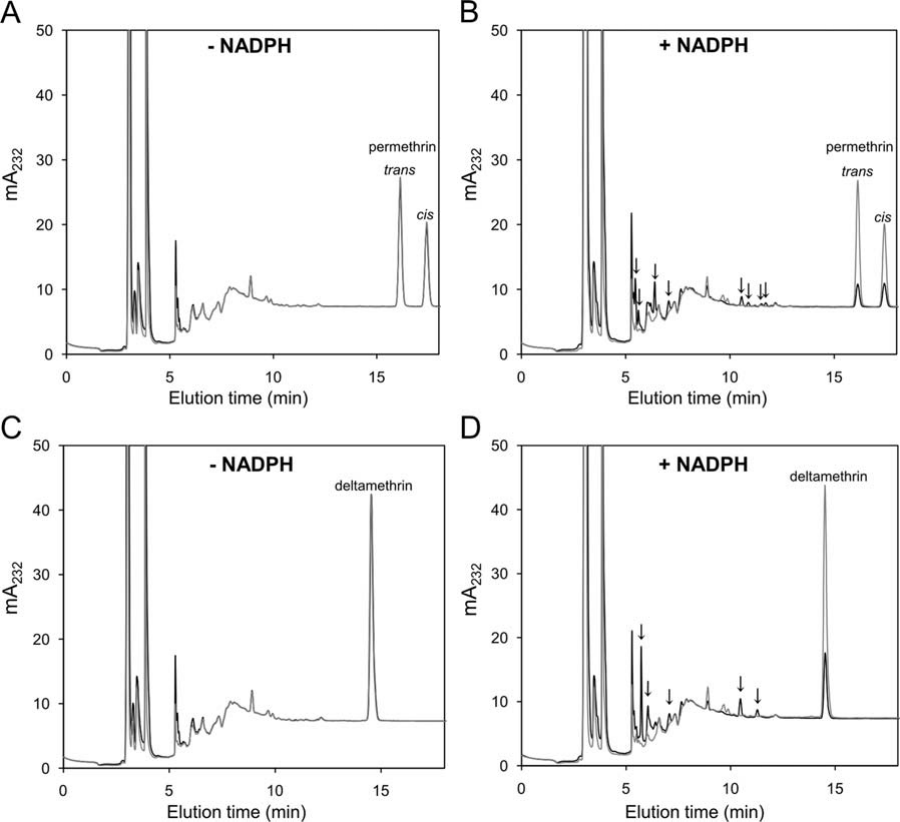
HPLC chromatograms of CYP6P3 reactions. (A) and (B) show CYP6P3 reactions with 10 µM permethrin. (C) and (D) show CYP6P3 reactions with 10 µM deltamethrin. Substrate peaks for *cis-* and *trans-*permethrin stereoisomers and deltamethrin are indicated. (B) and (D) are overlaid traces of reactions quenched after 0 min (light trace) and 30 min (dark trace) showing substrate elimination in the presence of NADPH. (A) and (C) are overlaid negative control reactions quenched after 0 and 30 min in the absence of NADPH. Putative NADPH-dependant metabolite peaks are indicated by arrows.

**Figure 5:**
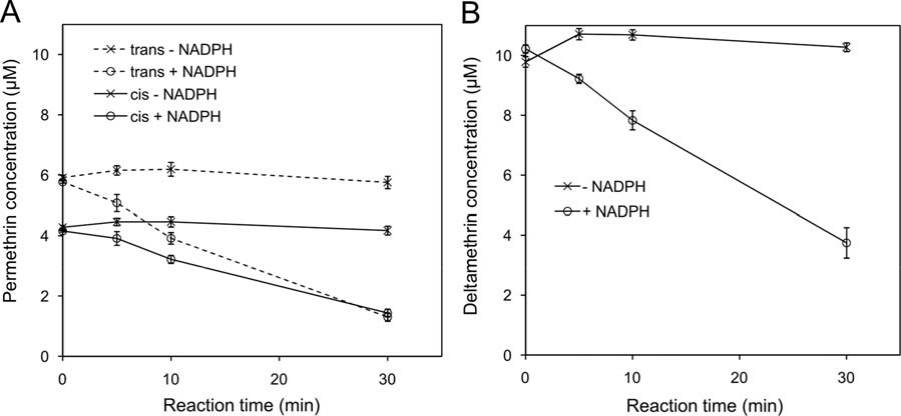
Time course of pyrethroid metabolism. (A) Reactions were performed at 30°C with 10 µM deltamethrin or (B) 10 µM permethrin stereoisomer mixture. Concentrations were determined by HPLC peak integration (Mean values±S.E.M., *N* = 3).

CYP6P3 was co-expressed with *A. gambiae* cytochrome P450 reductase (CPR) in *E. coli* to produce a functional monooxygenase complex, and the ability of CYP6P3 to metabolise permethrin was evaluated from time-dependant elimination of a 10 µM mixture of four isomers. Permethrin eluted with R and S *trans*-isomers at 16.1 min and R and S *cis*-isomers at 17.4 min in HPLC analysis. In the absence of NADPH there was no significant change in permethrin concentration over the 30 min incubation period (Figures 4A and 5A). With the NADPH regeneration system included, 72% of the total permethrin was eliminated in 30 min (Figure 4B) with a steady rate of elimination (Figure 5A). This indicates a turnover of 0.59±0.04 min^−1^, (slope from linear regression±S.E.M.) for the *trans*-permethrin isomers and 0.37±0.02 min^−1^ for the *cis*-permethrin isomers (combined rate of 0.97±0.06 min^−1^).

CYP6P3 activity was also tested against an alpha-cyano pyrethroid, deltamethrin, commonly used on insecticide-treated bednets. Deltamethrin eluted at 14.5 min and like permethrin, NADPH-dependent elimination by CYP6P3 was observed (Figures 4C, 4D, and 5B). The single isomer at 10 µM was turned over slightly slower than permethrin at a constant rate of 0.86±0.03 min^−1^. Deltamethrin metabolism was studied in greater kinetic detail due its availability as a single isomer. Analysis of initial metabolic rate (5 min reactions) in response to deltamethrin concentration revealed Michaelis-Menten kinetics: the V_max_ was 1.8±0.2 min^−1^ and the *K*_m_ was 5.9±1.2 µM (±S.E.M., *N* = 3).

## Discussion

In this study, we selected wild-caught mosquitoes from a highly permethrin resistant field population in southern Ghana against the insecticide permethrin. Using a custom made microarray we identified *CYP6P3*, a P450 that was overexpressed in mosquitoes surviving exposure to 0.75% permethrin for 2 h, the time that kills 50% of the mosquito population. Heterologous expression of CYP6P3 in *E. coli* yielded a protein that metabolises permethrin and deltamethrin. This is the first study to identify a gene encoding for an enzyme that mediates pyrethroid detoxification in the malaria vector *A. gambiae s.s.*. As our findings are based on the study of gene expression in wild-caught, phenotyped mosquitoes, the results are of significant importance in the field context.

3-day old females of the mosquito population under study showed 83% survival rate at the WHO diagnostic to 0.75% permethrin for 1 h. To our knowledge, this is the highest survival rate reported against permethrin in an *A. gambiae* field population to date. This population has a high frequency of the L1014F *kdr* allele, which confers resistance to pyrethroids and DDT [6]. The population is almost fixed with 91% of screened individuals found to be homozygous for the L1014F substitution at the this locus, a 12% increase compared to a survey conducted in 2002 at the same field site [18]. There has been much debate over the extent to which target-site and metabolic resistance mechanisms contribute to the observed phenotype [3],[7],[32]. To exclude any possible effect of known target-site mutations we performed all gene expression analyses only on RNA extracted from specimens homozygous for the L1014F *kdr* type. This is a considerable improvement over previous expression studies which were potentially confounded by comparing the susceptible Kisumu strain with resistant laboratory strains having either the L1014S [11],[13],[24] or L1014F [12] *kdr* mutation. Furthermore, in the field population studied L1014F allele was close to fixation and hence the observed variability in resistance phenotype is most likely attributable to an additional mechanism. The time-response, showing mortality as a function of exposure time to 0.75% permethrin, is represented by a very broad symmetrically shaped sigmoid curve. This implies that the population has a broad distribution of resistant phenotypes [33] which suggests that there are multiple resistance mechanisms present in the population.

Three P450s up-regulated in the permethrin-selected specimens showed good accordance between the microarray and RT-PCR data, including *CYP6P3*, *CYP6M2* and *CYP6AK1*. P450s are an abundant family of enzymes which can mediate resistance to all classes of insecticides and their up-regulation has been documented in a broad range of insect species [8],[9]. Although up-regulation has been identified for a large number of P450s in insecticide resistant insects, studies of catalytic activity are generally limited [9]. To date two *A. gambiae* P450s (*CYP6Z1* and *CYP6Z2*) have been functionally characterised [14],[15]. While CYP6Z1 is capable of metabolising DDT [14] and CYP6Z2 binds to pyrethroids, a catalytic capacity could not be shown for pyrethroids [15]. The current study focused on the characterisation of CYP6P3 because there was a strong association between gene expression and resistance phenotype. CYP6P3 is the first enzyme with a demonstrated potential for catalytic activity with pyrethroids in *A. gambiae*. Intriguingly, *CYP6P9*, the *A. funestus* ortholog of *CYP6P3* [34], is located within a major Quantitative Trait Locus (QTL) conferring pyrethroid resistance [35] and overexpressed in adults of the pyrethroid resistant FUMOZ-R strain [36]. As both the QTL marker and the *A. funestus CYP6P9* locus are physically mapped to the same region on chromosome 2R, it has been postulated that up-regulation is mediated via mutations in *cis*-acting elements [36]. In *A. gambiae s.s.* the question whether *CYP6P3* is *cis*- or *trans*-regulated remains unanswered and further studies are needed to identify how up-regulation is controlled. This information will facilitate the development of expression-associated DNA markers that would allow screening of wild populations for the presence of metabolic resistance alleles.

The second P450 which showed convincing evidence for association with permethrin-resistance was *CYP6M2*. Moreover, *CYP6M2* has previously been identified in a colonised laboratory strain from the same field site [12]. Enzyme characterisation of CYP6M2 is currently underway.

The third P450, *CYP6AK1*, has not previously been associated with pyrethroid resistance and was down-regulated in the DDT-resistant ZAN/U strain [11]. *CYP6AK1* has not been investigated further, but this gene may become an interesting candidate if found in future studies.

We expressed the full-length cDNA of CYP6P3 in *E. coli* along with its cognate redox partner CPR to produce a functional enzyme for characterisation studies. Consistent with a role in detoxification, CYP6P3 was found to metabolise permethrin. Permethrin consists of four isomers: (R) *cis*, (R) *trans*, (S) *cis*, (S) *trans*, and it is the *cis* isomers that has greater insecticidal activity, possibly due to slower metabolism [37]. Since two peak mixtures of *cis* R/S and *trans* R/S isomers are separated by HPLC chromatography, rates of metabolism of individual isomers could not be determined. However, both (1RS) *cis* and (1RS) *trans* isomers were eliminated from enzyme reactions indicating that metabolism of the active form occurs. Moreover the enzyme was efficient in metabolising deltamethrin, which is widely used in agriculture and in the production of insecticide-treated bednets, further emphasising a potentially important role in metabolic resistance.

Modest rates of metabolism of the pyrethroids by the heterologously expressed CYP6P3 were observed. Substrate turnover values were in the range 0.5–2 min^−1^, which were 5 to 10-fold slower than the rates observed for the *in vitro* P450 metabolism of pyrethroids reported from other species; the lepidopteran CYP6B8 has a V_max_ for α-cypermethrin of 13 min^−1^ [38] whereas rat CYP3A2 has 14-fold higher turnover than CYP6P3, although the K_m_ for deltamethrin is not significantly different [39]. This could potentially be due to the absence of cytochrome b_5_ in our system, which is known to enhance the activity of some P450s [8]. Indeed, increased levels of cytochrome b_5_ are associated with P450 mediated insecticide resistance in some insects and are directly involved in CYP6D1 mediated cypermethrin metabolism in the house fly [40]. Investigations are underway to examine the influence of cytochrome b_5_ on metabolism and to further define the molecular interactions of pyrethroids and other insecticides with CYP6P3.

Our data demonstrates that a P450, *CYP6P3* is up-regulated in highly permethrin resistant *A. gambiae s.s.* mosquitoes in the field and functional characterisation of the enzyme strongly suggest that CYP6P3 metabolises both permethrin and deltamethrin. The overexpression of its ortholog in *A. funestus* provides further support to the importance of this enzyme for pyrethroid resistance in malaria vectors. *CYP6M2* was also overexpressed in this study and a study on a laboratory strain colonised from the same area [12] and thus merits further investigation. Yet, although its origin is from the same locality as the existing population, the previous analysis did not detect the change in *CYP6P3*. The current study emphasises the importance of studying metabolic resistance in natural mosquito populations.

## Supporting Information

Figure S1 Transformation of fold differences for mixed RNA pools. (A) A simplified mathematical model that adjusts for limitations in the fold change of mRNA levels if RNA pools from insecticide-selected (*S*) *vs.* a mixed (insecticide-selected combined with unselected) group (*M*) of mosquitoes are compared. The transformed ratio, *S*/*D* gives the ratio as if RNA could be extracted from survivors (*S*) and dead (*D*) mosquitoes alike and would be directly compared, a situation which may not be possible for selection experiments due to post-mortem RNA degradation. The model may be applicable wherever mosquitoes are selected from a population/laboratory colony and then compared back to their “parental” group or strain. The function depends on the mortality rate which is given by the number of susceptible individuals in the selection experiment. (B) The graph plots the relationship between observed and “true” ratio for the mortality observed in this study (*m* = 0.58) and for a 25% and 75% mortality rate.
(0.32 MB TIF)
