# Supplementary figures and images for: Field-Caught Permethrin-Resistant Anopheles gambiae Overexpress CYP6P3, a P450 That Metabolises Pyrethroids
Source: PLoS Genet. 2008 Nov 28;4(11):e1000286. doi: 10.1371/journal.pgen.1000286 (PMC2583951; doi:10.1371/journal.pgen.1000286)

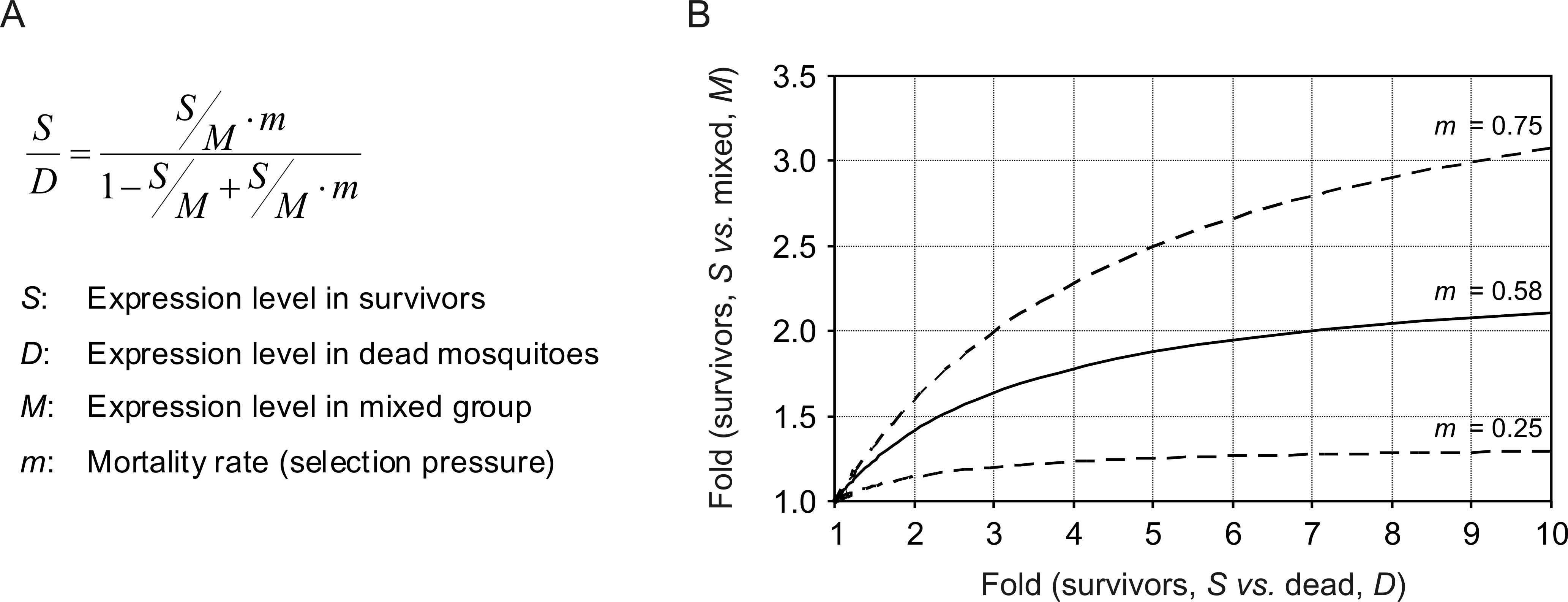

Supplement: Figure S1 — Transformation of fold differences for mixed RNA pools. (A) A simplified mathematical model that adjusts for limitations in the fold change of mRNA levels if RNA pools from insecticide-selected (S) vs. a mixed (insecticide-selected combined with unselected) group (M) of mosquitoes are compared. The transformed ratio, S/D gives the ratio as if RNA could be extracted from survivors (S) and dead (D) mosquitoes alike and would be directly compared, a situation which may not be possible for selection experiments due to post-mortem RNA degradation. The model may be applicable wherever mosquitoes are selected from a population/laboratory colony and then compared back to their “parental” group or strain. The function depends on the mortality rate which is given by the number of susceptible individuals in the selection experiment. (B) The graph plots the relationship between observed and “true” ratio for the mortality observed in this study (m = 0.58) and for a 25% and 75% mortality rate. (0.32 MB TIF) [file pgen.1000286.s001.tif]
